# Extranodal diffuse large B-cell lymphoma with monoclonal gammopathy: an aggressive and primary refractory disease responding to an immunomodulatory agent

**DOI:** 10.1186/s40164-015-0030-1

**Published:** 2016-01-06

**Authors:** Patrizia Mondello, Vincenzo Pitini, Valeria Barresi, Elliott Joseph Brea, Cristian Di Mirto, Carmela Arrigo, Salvatore Cuzzocrea, Michael Mian

**Affiliations:** 1 Department of Human Pathology, University of Messina, Via Consolare Valeria, 98100 Messina, Italy; 2 Department of Chemical, Biological, Pharmaceutical and Environmental Sciences, University of Messina, Messina, Italy; 3 Lymphoma Department, Lymphoma Service, Memorial Sloan Kettering Cancer Center, 1275 York Avenue, New York, NY USA; 4 Molecular Pharmacology and Chemistry Program, Sloan-Kettering Institute, New York, NY USA; 5 Weill Cornell Medical College, New York, NY USA; 6 Department of Internal Medicine, University of Messina, Messina, Italy; 7 Department of Hematology and CBMT, Ospedale di Bolzano, Bolzano, Italy; 8 Universitätsklinik für Innere Medizin V, Hämatologie and Onkologie, Innsbruck, Austria

**Keywords:** Diffuse large B-cell lymphoma, Monoclonal gammopathy, Serum free light chain, Lenalidomide

## Abstract

**Background:**

Although the clinical outcome of patients with diffuse large B cell lymphoma (DLBCL) has been improved by the addition of rituximab to standard chemotherapy, almost one-third fails or relapses after first line treatment. The presence of monoclonal gammopathy (MG) is a known adverse prognostic factor for DLBCL. Because this subset of patients does not benefit from R-CHOP, new therapeutic options are required. Herein, we report the first case of extranodal DBCL of the lung with a concomitant MG who achieved a long lasting complete remission with lenalidomide.

**Case presentation:**

The 73-year-old male patient presented with lateral cervical lymphadenopathy, B symptoms, lactate dehydrogenase and beta2-microglobulin elevation. Computed tomography (CT) showed mediastinal lymphadenopathy and bilateral lung involvement. Biopsy of both disease locations revealed the presence of DLBCL. Successive bone marrow trephine biopsy proved the presence of concordant DLBCL involvement. At the time of diagnosis, a MG was present as well. The patient did not respond to the standard treatments, and subsequently underwent lenalidomide 25 mg/m^2^ days 1–21 q28 plus dexamethasone 40 mg days 1–4, 9–12 e 17–20. This therapeutic regimen was efficacious and safe as salvage therapy in extranodal DBCL with a MG. Furthermore, we observed a close association between DLBCL response to therapy and MG levels, suggesting that the amount of M-protein might be a surrogate marker of disease response.

**Conclusion:**

Although DLBCL associated with MG does not respond properly to the standard treatments, it is highly sensitive to lenalidomide, which is why we endorse its role as treatment of choice in this subset of patients. In addition, MG levels appear to correlate with tumor burden, suggesting that it might be a useful marker of disease response. Prospective trials to validate these observations are warranted.

## Background

Diffuse large B-cell lymphoma (DLBCL) is the most common B-cell non-Hodgkin lymphoma (NHL), accounting for about 30 % of all new diagnoses [1]. Despite its typical morphology, it is a very heterogeneous disease consisting of many subtypes. Gene expression profiling (GEP) studies have identified three main DLBCL subgroups based on the cell of origin: germinal center B-cell (GCB), activated B-cell (ABC) and unclassified DLBCL being mostly represented by primary mediastinal B-cell lymphoma (PMBCL) [2, 3]. These three subtypes have distinct oncogenic driver pathways resulting in a different prognosis [4, 5]. In particular, ABC DLBCL patients have shown a worse survival than GCB DLBCL when treated with rituximab, cyclophosphamide, vincristine, doxorubicin and prednisone (R-CHOP) [6]. Another clinical parameter reflecting the heterogeneity of this disease is the presence of monoclonal gammopathy (MG) in some patients [7]. DLBCL with MG often shows an immunoblastic differentiation, however, due to its morphologic resemblance with plamosmobastic lymphoma (PBL), diagnosis can be controversial. Nevertheless, the immunophenotype of these diseases is different: DLBCL with MG usually express the pan-B-cell antigens [8] while PBL has a characteristic immunophenotypic pattern with cluster of differentiation (CD) 20 and CD 79a negativity in combination with markers of post germinal center B-cell and plasma cell, such as MUM1+, IgG+, CD 138+, CD 38+ [9, 10]. Furthermore, most of PBL develop in the setting of viral infection and/or immunodeficiency. In addition, a rare variant of PBL is characterized by having a translocation of ALK to the clathrin gene on chromosome 17 [11]. Even multiple myeloma (MM) with plasmobastic features present morphological similarities to DLBCL with MG. However, its immunophenotype resembles PBL [12–14]. Moreover, DLBCL with MG differ from MM with plasmobastic features by the absence of bone involvement with radiographically evident lytic lesions [15].

Usually, patients affected by DLBCL with MG present with advanced stage disease, involvement of extranodal sites and high IPI-score [16]. Clinical data suggest that DLBCL with MG has a very aggressive clinical course, not adequately responding to standard treatments, which is why alternative approaches are needed. Lenalidomide, an oral immunomodulatory drug (IMiDs) with multiple mechanisms of action, including direct antitumor and immunomodulatory effects [17], has demonstrated to be active as single agent in NHL [18] with a higher response rate in non-GCB with respect to GCB patients (55 vs 9 %, respectively) [19]. Nevertheless, lenalidomide represents the cornerstone of MM management, providing rapid and sustained disease control in relapsed/refractory [20, 21] as well as in newly diagnosed patients [22]. Based on its dual efficacy on lymphoma and on myeloma cells, lenalidomide might have a significant role in DLBCL with MG.

Herein, we present the first case of extranodal DBCL of the lung associated with a MG resistant to standard therapies but highly responsive to lenalidomide. Furthermore, we observed a close association between DLBCL response to therapy and MG serum levels, suggesting that in this specific situation the dosage of the MG might be a valid marker of disease response.

## Case presentation

**Fig. 1:**
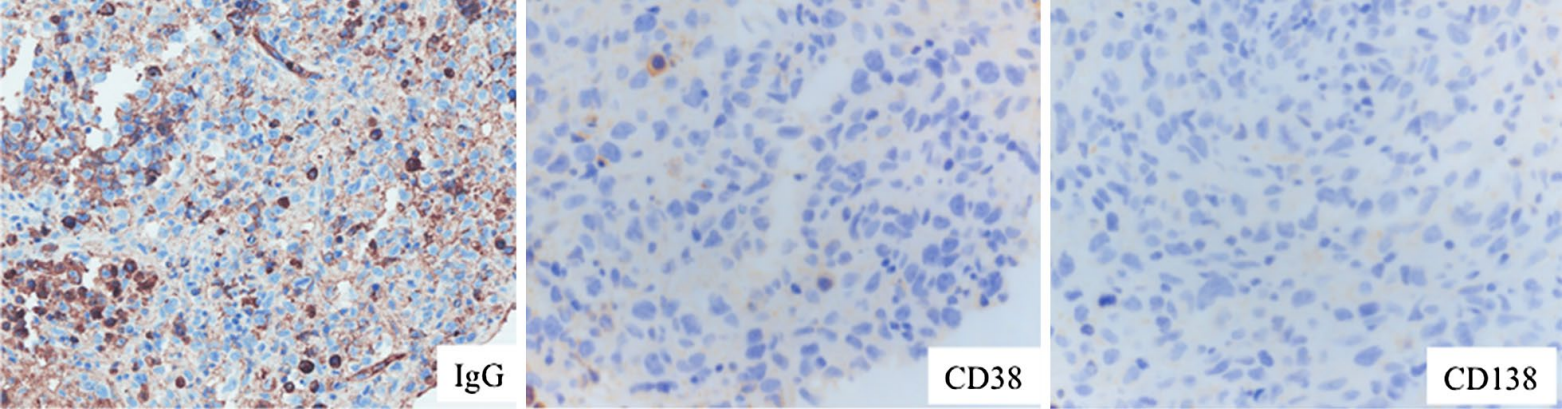
Diffuse large B-cell lymphoma (DLBCL) in lymph node biopsy. Immunohistochemistry revealed IgG staining in the cytoplasm of some neoplastic cells (IgG stain; original magnification, ×200). Absence of CD38 expression in neoplastic cells and a positive staining in intermingled plasma cells (original magnification, ×400). Absence of CD138 stain in neoplastic cells (original magnification, ×400)

**Fig. 2:**
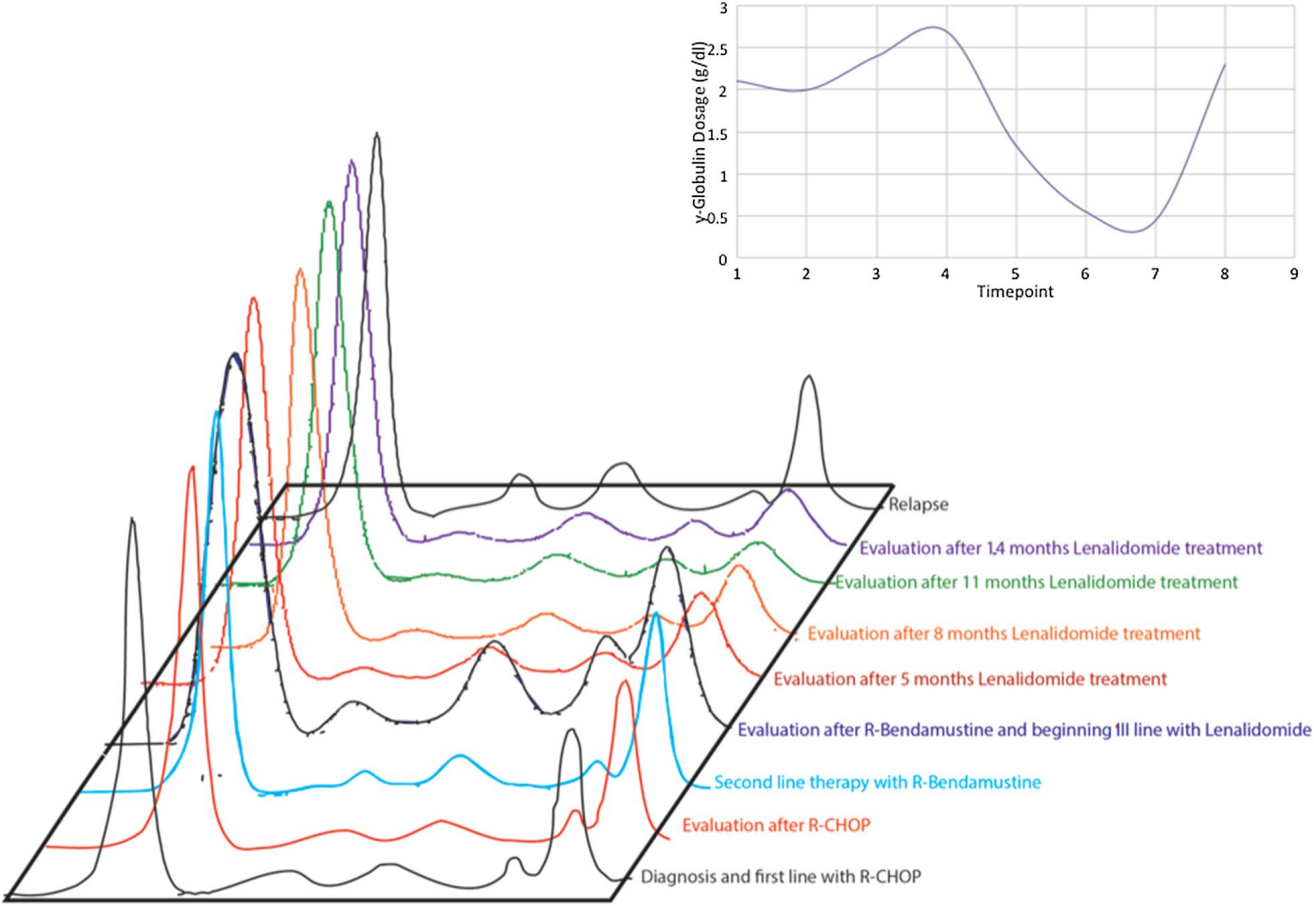
Trend of γ-globulin dosage during the disease course

**Fig. 3:**
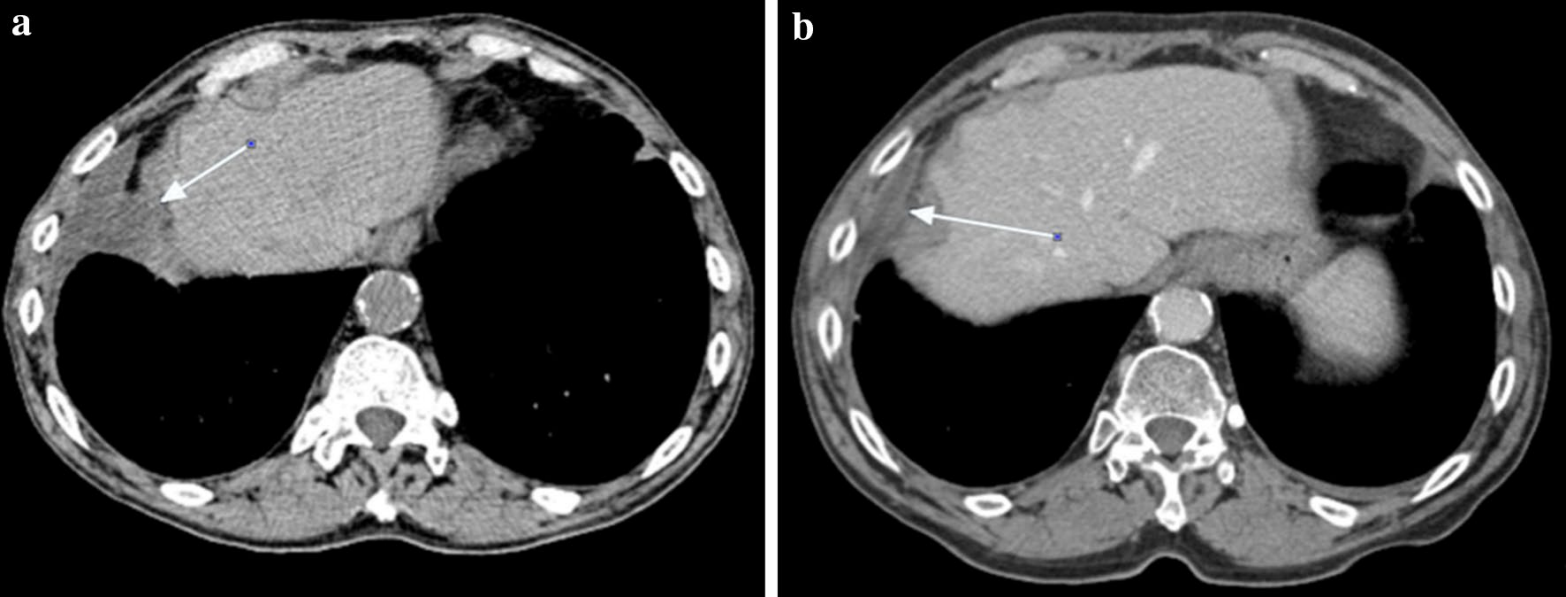
Computed tomography (CT) of the torax. **a** Pathological tissue with elongated morphology of maximum diameter of 6 cm. **b** Almost complete disappearance of the pathological mass previously observed

In August 2011, a 73 year-old man presented with lateral cervical lymphadenopathy, B symptoms and lactate dehydrogenase (LDH) and beta2-microglobulin (B2 M) elevation (890 U/L, upper normal 460 and 3176 ng/mL, upper normal 2150, respectively). Computed tomography (CT) revealed mediastinal lymphadenopathy and bilateral lung involvement. Histological examination of a lymph node and lung biopsies detected the presence of a DLBCL with an intermediate-high proliferation index (55 % Ki-67 positive cells) without plasmoblastic differentiation. In immunohistochemistry, the neoplastic tissue was CD 20+, CD 19+, CD 79 alfa+, CD 10+, CD 3−, CD 5−, CD 23+, CD 38−, CD138−, BCL2−, BCL6+, MUM1+, suggesting a non-GCB origin [23], and suggestive for IgG expression (Fig. 1). Bone marrow trephine biopsy proved the presence of concordant DLBCL involvement. The IPI score was high due to four risk factors (stage IV, age >60, elevated LDH and >1 extranodal site). At the time of diagnosis, a monoclonal gammopathy was accidentally discovered. The M-gradient was of immunoglobulin (Ig) G k-type, with a serum concentration of 22.5 g/dL. Serum λ free light chains (FLC), the same subtype as expressed by the neoplastic cells, were elevated (410.3 mg/l; range 5.5–355.2) resulting in an abnormal κ/λ FLC ratio. All the other Ig levels were normal (IgA 3.32—normal range 0.82–4.76 g/dL, IgM 1.84—normal range 0.30–2.27). The Bence-Jones proteinuria was absent. The concomitant presence of a multiple myeloma was excluded since the bone marrow plasmacytosis was less than 5 % and the patient had no end-organ damage according to the CRAB criteria [24]. Since the patient was in a good clinical condition (performance status of 1), he underwent R-CHOP. Due to stable disease assessed by CT scan after the fourth cycle treatment was discontinued. At this time the MG remained stable (Fig. 2). Six months later the patient suffered a histological-proven progression in the lung. Due to a poor performance status (PS 2) at the time of relapse and his ineligibility to ASCT, the patient underwent bendamustine 100 mg/m^2^ days 1–2 q28 plus rituximab 375 mg/m^2^ every 28 days for four cycles. Disease restaging after treatment completion revealed progressive disease (PD) (Fig. 3) as well as an increase of the MG (Fig. 2). Due to the lack of other treatment options, he finally underwent lenalidomide 25 mg/m^2^ days 1–21 q28 thereafter. Dexamethasone 40 mg days 1–4, 9–12 e 17–20 for the first four cycles and only days 1–4 was added as well. This because dexamethasone has demonstrated activity as single agent in reducing paraproteinemia and improving quality of life [25, 26]. Moreover, it synergistically inhibits tumor growth and induces apoptosis when combined with lenalidomine [27].

Hematologic toxicity was limited to reversible grade two neutropenia and thrombocytopenia after the sixth cycle. Except for fatigue grade two, no extra-hematologic toxicities were observed. Two months after treatment start, the patient’s general conditions improved and after 5 months he achieved a complete remission (CR) (Fig. 3). For the first time paraprotein was no longer detectable and immunofixation (IF) was negative as well (Fig. 2). Overall, the patient underwent 15 cycles of lenalidomide until he suffered relapse leading to death. Interestingly, the reappearance of a monoclonal IgG and of FLC k/l abnormal ratio preceded relapse (Fig. 2).

Herein, we report the case of a patient with DLBCL of the lung associated with the presence of a sieric MG of IgG λ type. Despite the histologic morphology not being compatible with a plasmablastic differentiation, the levels of MG following lymphoma response and the absence of multiple myeloma features suggest that the paraprotein was produced by the lymphoma cells. Moreover, neoplastic cells represented with a λ light chain restriction the same subtype of light chain as the aberrant MG. As previously reported by others, MG was associated with a non-GCB subtype [28]. This could probably be explained by the fact that ABC DLBCL develops from post-germinal center B-cells and is associated with an up-regulation of Blimp-1, which is a master regulator of plasma cell differentiation [29]. Consistent with previous reports [9, 28], the presence of MG was associated with advanced stage, involvement of extranodal sites, elevated LDH and high IPI score. Despite this usually unfavorable risk profile at diagnosis, data regarding the outcome of such patients are still conflicting. Maurer et al. [30] showed that a monoclonal elevation of FLC ratio was only moderately associated with DLBCL outcome, while more recent analyses have observed a poor outcome in DLBCL with paraproteinemia [28, 31], especially in the ABC subtype [7].

As also observed by others, the benefit from R-CHOP in patients with DLBCL with a serum MG is very limited [7, 16] and new therapeutic options are required. Lenalidomide has demonstrated to be highly active in non-GCB [29] as well as in MM [22]. Recent insights into the biology of IMiDs have identified cereblon, a substrate-recognition component of an ubiquitin ligase [32], as a crucial molecule for the immunomodulatory and antiproliferative activities of lenalidomide [33, 34]. Lenalidomide-bound cereblon acquires the ability to target the proteosomal degradation of two B-cell transcription factors, IKZF1 and IKZF3, an essential step in the anti-myeloma effect [35]. In addition, lenalidomide induces the cereblon-mediated down-regulation of IFR4 leading to inhibition of the B-cell receptor-NF-kB signaling pathway [34], which is aberrant in ABC DLBCL [2]. Despite breakthrough studies that identified cereblon as a critical lenalidomide target [32, 33], there are challenges in its use as a biomarker. In particular, no correlation between cereblon gene expression or protein level to sensitivity or to intrinsic resistance to lenalidomide treatment has been observed [36, 37]. Therefore, the clinical value of cereblon expression as a predictive or prognostic biomarker is still questionable.

In our case, despite inefficacy of R-CHOP and R-bendamustine, lenalidomide induced a complete response with the disappearance of paraproteinemia. This striking efficacy might be related to its dual activity on lymphoma and on plasma cells, allowing to overcome the adverse effect connected to ABC DLBC and MG. Moreover, the favorable toxicity profile of lenalidomide translates into a high feasibility since ABC DLBC with MG are more common among old age patients [7]. Interestingly, we observed a direct correlation between DLBCL response to treatment and MG serum levels, suggesting that the aberrant proteins were produced by the neoplastic cells. Consequently, the monoclonal component might be used as a marker of disease in such cases.

## Conclusions

In conclusion, these data suggest that DLBCL associated with monoclonal gammopathy does not respond properly to the standard treatments while it could be highly sensitive to lenalidomide. The IMID was able to overcome the negative impact of MG and ABC DLBCL on response, which is why lenalidomide might be the treatment of choice for this subset of patients. In addition, MG levels appear to correlate with tumor burden, suggesting that it might be a useful marker of disease response. Prospective trials to validate these observations are warranted.

## Consent

Written informed consent was obtained from the patient for the publication of this report and any accompanying images.

## Abbreviations

DLBCL: diffuse large B cell lymphoma
NHL: B-cell non-Hodgkin lymphoma
GCB: germinal center B-cell
ABC: activated B-cell
PMBCL: primary mediastinal B-cell lymphoma
R-CHOP: rituximab, cyclophosphamide, vincristine, doxorubicin and prednisone
MG: monoclonal gammopathy
IMiDs: immunomodulatory drug
MM: multiple myeloma
LDH: lactate dehydrogenase
B2M: beta2-microglobulin
CD: cluster of differentiation
Ig: immunoglobulin
FLC: free light chains
PD: progressive disease
CR: complete remission
IF: immunofixation
